# Effects of metformin on arterial elasticity and pro-inflammatory markers in black diabetes patients

**DOI:** 10.4102/hsag.v29i0.2419

**Published:** 2024-06-14

**Authors:** Tsakani L. Rasakanya, Elzbieta Osuch

**Affiliations:** 1 Department of Pharmacology and Therapeutics, School of Medicine, Sefako Makgatho Health Science University, Pretoria, South Africa

**Keywords:** arterial elasticity, pulse wave velocity, augmentation index, type 2 diabetes mellitus, pro-inflammatory markers

## Abstract

**Background:**

Pro-inflammatory markers are linked with the development and progression of type 2 diabetes mellitus and arterial stiffening. Pulse Wave Velocity (PWV) and Augmentation Index (Aix) are non-invasive standard markers of arterial elasticity and predictors of cardiovascular mortality and morbidity.

**Aim:**

To investigate the effects of metformin alone and in combination with glimepiride on arterial elasticity, pro-inflammatory cytokines in black type 2 diabetes mellitus patients.

**Settings:**

Participants were enrolled from Sefako Makgatho Health Sciences University community, Gauteng, South Africa.

**Methods:**

PWV and Aix were measured using the AtCor SphygmoCor^®^ system (AtCor Medical, Inc., Sydney, Australia). Cytokines levels were measured using Multiplexing with Bio-Plex Pro™ human inflammation panel I assay. Treatment naïve type 2 diabetes participants were divided into two groups: metformin (M) (*n* = 10) and metformin glimepiride (MS) (*n* = 14). The study participants were followed up at 4 and 8 months after treatment initiation.

**Results:**

In the M and MS, IL-1β increased significantly at four months (58.19 ± 0.03 pg/ml, 58.35 ± 0.30 pg/ml) when compared to baseline (33.05 ± 18.56 pg/ml, 34.79 ± 18.77 pg/ml) then decreased significantly at eight months (29.25 ± 11.64 pg/ml, 32.54 ± 14.26 pg/ml) when compared to four months (58.19 ± 0.03 pg/ml, 58.35 ± 0.3 pg/ml) (*p* < 0.05). There were no significant changes in PWV, Aix, IL-1ra, IL-2, IL-6, IL-8, TNF-α and hs-CRP levels at both treatment intervals.

**Conclusion:**

Metformin alone or in combination with glimepiride did not improve arterial elasticity and did not reduce pro-inflammatory cytokines levels in T2DM black South African patients.

**Contribution:**

The context-based knowledge generated by the current study is expected to enhance the continuum of care for T2DM patients.

## Introduction

Arterial stiffening reflects a steady fragmentation and loss of elastin and build-up of stiffer collagen fibres in the arterial wall. Over time, the walls of large arteries particularly the aorta lose elasticity which results in increased arterial stiffness (Oh 2018). Structural, cellular and molecular modifications are processes which the arterial wall is continuously subjected to. These processes result in changes of the wall structure, dimensions, contractile and elastic properties; they involve cellular growth properties, apoptosis, cell migration, inflammation and fibrosis (Dietrich et al. 2010).

Pulse wave velocity (PWV) and augmentation index (Aix) are regarded as the gold standard non-invasive methods for measuring arterial stiffness. PWV can be defined as the distance travelled by the pulse wave divided by the time taken to travel the distance and can be measured at any arterial segments between two regions. Increased speed of the pulse wave in the artery indicates increased arterial stiffness. Carotid-femoral PWV is considered as the gold standard for measuring central arterial stiffness (Franklin 2008; Lee & Oh 2010; Osuch et al. 2012). Augmentation index is an indirect measure of arterial stiffness. It is a measure of pulse wave reflection that computes how much of the central pulse pressure that is accounted for by the reflected pulse wave (Janner et al. 2012). The timing and amplitude of the reflected wave eventually depend on the stiffness of the small vessels and large arteries and therefore, Aix provides a measure of systemic stiffness (Wilkinson et al. 2002).

Studies have shown that type 2 diabetes mellitus (T2DM) is a pro-inflammatory state with the evidence of increased levels of high-sensitivity C-reactive protein (hs-CRP) and pro-inflammatory cytokines: interleukin 1β (IL-1β), interleukin 6 (IL-6) and tumour necrosis factor alpha (TNF-α), among others (Misra, Das & Sahu 2012). Type 2 diabetes mellitus is characterised by several grades of insulin resistance and relative deficiency in its production. Insulin resistance has been accredited to adipose tissue activation linked with an elevated release of inflammatory cytokines such as TNF-α and IL-6 (Cruz et al. 2013).

Metformin use may decrease cardiovascular diseases (CVD) via an effect on arterial stiffness, and it has been proposed that metformin interferes with the formation of advanced glycation end products (AGEs) thereby inhibiting cross-linkage of the pressure load-bearing elements of the arterial wall by AGEs (Van Bortel et al. 2012). Metformin was shown to act as an anti-inflammatory by suppression of nuclear factor kappa-light-chain-enhancer of activated B cells (NFĸB) via AMPK-dependent pathways in human vascular smooth muscle cells (VSMCs) (Lunder, Janić & Šabovič 2021). In a study done by Isoda et al. (2006), metformin inhibited IL-1β, IL-6 and IL-8 release from human vascular smooth muscle cells, endothelial cells and macrophages, and it also suppressed cytokine-induced NF-κB-dependent gene transcription (Zheng et al. 2012). By controlling hyperglycaemia, weight gain and lipid profile, metformin exhibits anti-inflammatory action indirectly resulting in a favourable effect on chronic inflammation (Saisho 2015).

The findings of the Maastricht Study demonstrated that metformin use had no beneficial effects on arterial stiffness in T2DM Caucasian patients (Driessen et al. 2019). However a study by Matsumoto et al. (2004) reported that metformin therapy attenuates the progression of carotid arterial wall thickness in T2DM Japanese patients (Matsumoto et al. 2004). Araki et al. (2006) showed that in T2DM patients recruited from Center of Osaka City University Hospital, metformin improved arterial elasticity (Araki et al. 2006); furthermore, a study by Shargorodsky et al. (2012) conducted at the Wolfson Medical Center in Israel showed that metformin treatment was linked with significant decrease in Aix after 1 year of treatment, suggesting that metformin may facilitate its vascular effects via glycaemic control-independent mechanisms (Shargorodsky et al. 2012).

The effects of metformin on arterial elasticity and pro-inflammatory cytokines in black type 2 diabetes South African patients have not been reported. The aim of this study was to investigate the effects of an 8-month treatment with metformin alone and in combination with glimepiride on arterial elasticity, pro-inflammatory cytokines (IL-1β, IL-6, IL-8, TNF-α and hs-CRP) and anti-inflammatory cytokines (IL-1ra, IL-2) in black T2DM patients.

## Research methods and design

Comparative study model was used. Consent was acquired from the participants before entering the study. Full explanation of the study procedures regarding measuring of vital signs, PWV and Aix, blood drawing via vacupunture for HbA1c, plasma glucose determination and analysis of cytokines was given with the possibility of withdrawal at any time. The study was accomplished in accordance with the principles detailed by the Declaration of Helsinki.

Twenty-four newly diagnosed treatment naïve type 2 diabetes participants were enlisted from Sefako Makgatho Health Sciences University community and Dr George Mukhari Academic Hospital. A global e-mail of the study information leaflet was sent to the university community, and face-to-face recruitment of the hospital patients was done. After screening, participants were divided into two groups. Participants were either treated with 850 mg metformin daily (M group) (*n* = 10) or with 850 mg plus 2 mg glimepiride daily (MS group) (*n* = 14). The study participants were followed up twice at 4 months and 8 months after treatment initiation. Participants with diabetes were not given additional medication.

Informed written consent was obtained using a consent document, after explaining the research and assessing participant comprehension. Questionnaires completed, vital signs and body mass index (BMI) were measured. Venous blood was collected via vacupunture after overnight fasting, and determination of HbA1c levels, plasma glucose and the high sensitivity C-reactive protein was done by the National Health Laboratory Services, a South African National Accreditation System (SANAS)-ascribed laboratory. To ensure the correct and consistent sample collection and handling, the National Health Laboratories Services handbook on standard operating procedures, version 1, active from 06 March 2015, and document number GPQ0064 were used as guidelines. The cytokine levels were measured using Multiplexing with Bio-Plex Pro human inflammation assays (Bio-Plex Pro™ human inflammation panel I). The concentrations of IL-1β, IL-1ra, IL-2, IL-6, IL-8 and TNFα were determined according to the manufacturer’s protocol.

Pulse wave velocity and Aix were noninvasively measured using the AtCor SphygmoCor^®^ system (AtCor Medical, Inc., Sydney, Australia). Electrocardiogram-gated carotid and femoral waveforms were documented using applanation tonometry. Carotid-femoral path length was calculated as the difference between the surface distance joining the (1) suprasternal notch, the umbilicus, and the femoral pulse and (2) the suprasternal notch and the carotid pulse. The carotid-femoral transit time was projected in 8–10 sequential femoral and carotid wave forms as the average time difference between the inception of the femoral and carotid waveforms. Pulse wave velocity was assessed as the carotid-femoral path length divided by the carotid-femoral transit time as expressed in metres per second (m/s).

Augmentation index was determined by pulse wave analysis (PWA). Radial artery waves were noninvasively recorded by applanation tonometry. Twenty waves were captured, and PWA was used to obtain a central aortic pulse wave and haemodynamic measures by a generalised authorised mathematical transfer function. Aix measurements were standardised to a pulse rate of 75 beats per min and extracted as a percentage (%). To identify the cytokines that were individually correlated with PWV and Aix, Pearson’s correlation coefficient analysis including all study participants (*n* = 24) was performed.

Sample size estimates were based on estimation of the difference in PWV between any two groups. With a sample size of 12 per group at the 5% level, a two-sided T-test will have 80% power to distinguish a difference of 2 m/s (7–9) in PWV between any two groups, assuming a common standard deviation of 2.5 m/s. Sample size estimation was done on a Query Advanced (Statistical Solution Ltd, Cortc, Ireland), version 8.1.1.0.

Validity was upheld by strict adherence to the inclusion and exclusion criteria. The AtCor SphygmoCor^®^ used for evaluating PWV and Aix was to be operated by a trained dedicated operator. All the equipment used in the lab investigations were purchased from reputable suppliers, and the procedures were performed according to the manufacturer’s protocol.

## Statistical analysis

All statistical analyses were done on SAS (SAS Institute Inc, Carey, NC, USA). Continuous variables were plotted as standard deviations and mean values. Mean values of the demographic variables were compared between the two test groups by analysis of variance (ANOVA) followed by pair-wise T-test comparison. Median values for both PWV and Aix were determined. Linear regression analysis was done with PWV and Aix @75 as outcomes (dependent) variables and the demographic variables as predictors (independent) variables.

### Ethical considerations

Ethical clearance to conduct this study was obtained from the Sefako Makgatho University Research Ethics Committee (No. SMUREC/M/112/2016:PG).

## Results

Of the 24 study participants, 11 were females and 13 were males aged 52.58 ± 12.7 years. The haemodynamic and clinical descriptions of the study participants are shown in Table 1. In the metformin group, BMI significantly increased at 4 months of treatment (29.90 ± 5.6 kg/m^2^) and 8 months (30.25 ± 5.6 kg/m^2^) from baseline (28.94 ± 5.44 kg/m^2^) (*p* < 0.05).

**TABLE 1:** Effect of metformin and glimepiride on study variables at baseline, 4 months and 8 months of treatment (*N* = 24).

Variables	M0 (*n* = 10)	MS0 (*n* = 14)	M1 (*n* = 10)	M2 (*n* = 10)	MS1 (*n* = 14)	MS2 (*n* = 14)
BMI	28.94 ± 5.44	31.42 ± 5.78	29.90 ± 5.6†	30.25 ± 5.6†	33.03 ± 4.5	33.55 ± 4.3
MAP	103.80 ± 16.80	103.14 ± 6.37	98.40 ± 9.1	100.60 ± 9.8	94.71 ± 12.3‡	103.50 ± 11.6
aPP	47.00 ± 24.70	38.00 ± 13.86	42.70 ± 17.2	38.40 ± 9.4	38.00 ± 15.1	44.36 ± 12.9
rPP	55.50 ± 21.77	38.00 ± 13.86	51.20 ± 16.9	48.80 ± 14.0	46.50 ± 14.8	55.86 ± 13.7
HR	73.20 ± 15.89	74.86 ± 12.15	66.60 ± 7.1	76.90 ± 8.1§	73.79 ± 17.9	72.64 ± 12.3
aSBP	125.20 ± 26.07	123.07 ± 10.77	122.50 ± 17.7	122.40 ± 11.4	114.57 ± 19.8	128.36 ± 17.1
aDBP	84.20 ± 11.38	85.14 ± 7.06	79.80 ± 4.9	84.00 ± 7.8	77.29 ± 7.9‡	83.79 ± 9.9
rSBP	133.90 ± 26.11	132.79 ± 11.60	129.90 ± 16.6	132.00 ± 15.9	123.07 ± 19.1	138.64 ± 17.74
rDBP	82.80 ± 11.35	83.57 ± 7.25	78.70 ± 5.12	83.20 ± 7.3	75.50 ± 7.5‡	82.64 ± 82.6
P glucose	9.19 ± 3.62	12.48 ± 7.57	8.90 ± 6.6	6.38 ± 2.9	6.98 ± 4.1‡	9.86 ± 5.9
HbA1c	8.68 ± 3.16	6.71 ± 2.62	8.62 ± 2.5	8.62 ± 2.5	9.58 ± 5.5	9.58 ± 5.5
PWV	7.86 ± 1.91	6.89 ± 5.37	8.34 ± 3.06	7.08 ± 1.87	6.53 ± 2.21	8.61 ± 3.50
Aix	28.60 ± 7.63	28.14 ± 8.44	27.90 ± 7.39	25.10 ± 9.48	24.53 ± 14.67	26.07 ± 11.70

Note: M0, M1, M2 metformin group visit 0 (baseline), visit 1 and 2 (4 and 8 months), MS0, MS1, MS2 metformin plus glimepiride visit 0 (baseline), visit 1 and 2 (4 and 8 months).

† , *p* < 0.05 M0 compared to M1, M2;

‡ , *p* < 0.05 MS0 compared to MS1, MS2;

§ , *p* < 0.05 M1 compared to M2; *p* < 0.05 MS1 compared to MS2.

BMI, body mass index; MAP, mean arterial pressure; aPP, arterial pulse pressure; rPP, radial pulse pressure; HR, heart rate; aSBP, aortic systolic blood pressure; rSBP, radial systolic blood pressure; aDBP, aortic diastolic blood pressure; rDBP, radial diastolic blood pressure; P, glucose plasma glucose; HbA1c glycated haemoglobin; PWV, pulse wave velocity; Aix, augmentation index.

In the metformin and glimepiride group, mean arterial pressure (MAP) significantly decreased at 4 months (94.71 ± 12.31 bpm) from baseline (103.14 ± 6.37 bpm) (*p* < 0.05). Arterial and radial diastolic blood pressure (DBP) were significantly decreased at 4 months (77.29 ± 7.91 mmHg) from baseline (85.14 ± 7.06 mmHg) (*p* < 0.05). Plasma glucose was significantly decreased at 4 months (6.98 ± 4.1 mmol/L) when baseline (12.48 ± 7.57 mmol/L) (*p* < 0.05).

There was no statistically significant difference in the effect of metformin alone and metformin plus glimepiride on PWV and Aix at 4 and 8 months of treatment (Table 1).

In the M and MS groups, IL-1β increased significantly at 4 months of treatment (58.19 ± 0.03 pg/mL, 58.35 ± 0.30 pg/mL) when compared to baseline (33.05 ± 18.56 pg/mL, 34.79 ± 18.77 pg/mL), then decreased significantly at 8 months (29.25 ± 11.64 pg/mL, 32.54 ± 14.26 pg/mL) when compared to 4 months (58.19 ± 0.03 pg/mL, 58.35 ± 0.3 pg/mL) (*p* < 0.05) (Table 2).

**TABLE 2:** Effect of metformin alone and metformin plus glimepiride on cytokines at 4 and 8 months of treatment compared to baseline (*N* = 24).

Cytokines	M0 (*n* = 10)	MS0 (*n* = 14)	M1 (*n* = 10)	M2 (*n* = 10)	MS1 (*n* = 14)	MS2 (*n* = 14)
**Pro-inflammatory cytokines**						
IL-1β	33.05 ± 18.56	34.79 ± 18.77	58.19 ± 0.03†,‡	29.25 ± 11.6	58.35 ± 0.3†,‡	32.54 ± 14.3
IL-6	21.70 ± 8.14	27.07 ± 9.35	21.70 ± 8.1	30.95 ± 21.7	28.07 ± 8.4	29.00 ± 17.8
IL-8	122.1 ± 164.6	112.2 ± 93.9	122.1 ± 164.6	418.2 ± 808.6	115.28 ± 93.9	360.2 ± 675.9
TNF-α	59.50 ± 26.15	69.25 ± 27.4	59.50 ± 26.2	55.45 ± 28.4	67.26 ± 27.4	62.93 ± 24.7
hs-CRP	10.30 ± 8.91	7.14 ± 9.07	10.00 ± 9.7	10.00 ± 9.7	7.43 ± 6.3	7.43 ± 6.3
**Anti-inflammatory cytokines**						
IL-Ira	38.70 ± 43.36	31.32 ± 17.84	38.70 ± 43.4	28.80 ± 18.6	33.32 ± 16.8	33.93 ± 20.5
IL-2	14.90 ± 7.94	18.39 ± 9.46	14.90 ± 7.9	15.70 ± 4.05	19.39 ± 9.7	15.54 ± 4.6

Note: M0, M1, M2 metformin group visit 0 (baseline), visit 1 and 2 (4 and 8 months), MS0, MS1, MS2 metformin plus glimepiride visit 0 (baseline), visit 1 and 2 (4 and 8 months).

† , *p* < 0.05 M0 compared to M1, M2; *p* < 0.05 MS0 compared to MS1, MS2;

‡ , *p* < 0.05 M1 compared to M2; *p* < 0.05 MS1 compared to MS2.

IL, interleukin; hs-CRP, highly sensitive C-reactive protein; TNFα, tumour necrosis factor alpha.

There were no statistically significant changes in IL-1ra, IL-2, IL-6, IL-8, TNF-α and hs-CRP levels at 4- and 8-months treatment.

Pulse wave velocity was directly correlated to IL-1β (r^2^ = 0.17800; *p* = 0.0262), IL-2 (r^2^ = 0.19659; *p* = 0.0139) and inversely correlated to IL1ra (r^2^ = –0.15251; *p* = 0.0544). Augmentation index was directly correlated to IL-6 (r^2^ = 0.15473; *p* = 0.0538) (Table 3).

**TABLE 3:** Pearson’s correlation coefficients of study cytokines with pulse wave velocity and augmentation index (*N* = 24).

Parameter estimates ± SE	PWV		Aix	
	r	*P*	r	*P*
IL-1β	0.17800	0.0262†	0.08660	0.2824
IL-6	0.01547	0.8480	0.15473	0.0538*
IL-8	−0.09622	0.2321	0.04716	0.5588
TNF-α	−0.09035	0.2620	−0.04078	0.6132
hs-CRP	0.05384	0.4620	0.12765	0.1055
IL-Ira	−0.15251	0.0544†	−0.07739	0.3369
IL-2	0.19659	0.0139†	0.13714	0.0878

IL, interleukin; hs-CRP, high sensitivity C-reactive protein; TNFα, tumour necrosis factor alpha; PWV, pulse wave velocity, AIX, augmentation index.

† , *p* < 0.05.

## Discussion

Prolonged low-grade inflammation plays a vital role in T2DM pathogenesis. Such a systemic and subclinical inflammatory process can be categorised by high levels of circulating inflammatory cytokines including hs-CRP, IL-1, IL-6, IL-8 and TNF-α. Several mechanisms have been indicated for cytokines in the development of T2DM development among some is that cytokines can directly block insulin receptor signalling by triggering c-Jun amino-terminal kinase and an inhibitor of nuclear factor kappa-beta kinase, resulting in serine phosphorylation of insulin receptor substrate (Pickup 2004).

The results of this study showed that PWV and Aix were directly correlated to pro-inflammatory cytokines (IL-1β, IL-6) and inversely correlated to anti-inflammatory cytokines (IL-1ra); however, metformin alone and in combination with glimepiride did not improve PWV and Aix over a period of 8 months of treatment and did not reduce pro-inflammatory cytokine levels. All participants had poor glycaemic control, which might have influenced the study outcomes. Hyperglycaemia is characterised by elevated levels of serum acute phase proteins and many cytokines (such as TNFα, lL-6, lL-1β) and can trigger monocytes to produce pro-inflammatory cytokines and chemokines. This modifies insulin sensitivity and consequently affects glucose metabolism (Juan, Minglian & Xingping 2014). Furthermore, chronic hyperglycaemia initiates the secretion of a damage-related S100A8 molecule (calgranulin A) from pancreatic islets that in turn increase macrophage infiltration leading to the production of pro-inflammatory cytokines, including IL-1β (Daryabor et al. 2020). IL-1β is a potent pro-inflammatory cytokine and is strictly regulated by IL-1ra. The balance in IL-1β and IL-1ra levels is critical in defining the response of β-cells and eventually the progression of T2DM. IL-1β facilitates insulin secretion and promotes β cell apoptosis, which can ultimately result in the development of T2DM (Alfadul, Sabico & Al-Daghri 2022).

These results are like those shown by Chang et al. (2018) where in uncontrolled T2DM patients, short-term treatment with oral hypoglycaemic drugs showed no significant improvement in the PWV, aortic augmentation index or markers of inflammation (Chang et al. 2018). However, these results contradict the results where metformin was shown to reduce the expression of IL-1β (Zak et al. 2020), IL-6 (Kang et al. 2017), IL-8 (Takemura et al. 2007), TNF-α (Hyun et al. 2013) and hs-CRP (Karbalaee-Hasani et al. 2012). Zak et al. (2020) showed that after 3-month treatment with metformin, IL-1β levels decreased by 88% and that the degree of its change was linked with the values of HbA1c. Inhibition of IL-1β leads to a decrease in the inflammatory process in pancreatic islets, and a reduction in glucose levels which results in an improvement of vascular complications and prevents the conversion of prediabetes to clinically expressed T2DM (Furmanova et al. 2019). IL-1β triggers the production of IL-6 and IL-8, and metformin inhibits IL-8 release from human adipose tissue in vitro. Other studies have shown that metformin suppresses IL-1β-induced production of IL-6 and IL-8 in human vascular wall cells (Takemura et al. 2007). A study by Hyun et al. (2013) showed that metformin inhibited the production of pro-inflammatory cytokines (IL-1β, IL-6, and TNF-α) by suppressing protein and mRNA expression in a dose-dependent manner. Tumour necrosis factor alpha is significant cytokine that influences not only inflammation but also metabolic diseases. Elevated TNF-α leads to insulin resistance by blocking insulin signalling pathways and altering the expression of glucose transporter 4 (Hyun et al. 2013). A systemic review and meta-analysis by Karbalaee-Hasani et al. (2021) indicated a significant decrease in circulating levels of CRP, TNF-α and IL-6 after metformin treatment in a subgroup of treatment duration ≥ 24 weeks (Karbalaee-Hasani et al. 2021).

Inflammation has been identified as the main trigger of T2DM (Ellulu & Samouda 2022), and T2DM accelerates the process of arterial stiffening (Rasakanya & Osuch 2023). The results of this study showed that PWV and Aix were directly correlated to pro-inflammatory cytokines (IL-1β, IL-6) and inversely correlated to anti-inflammatory cytokines (IL-1ra). Studies suggest that metformin exhibits its anti-inflammatory actions by inhibiting NFκB and activating AMPK (Saisho 2015). However, in this study, a lack of adequate glucose level control could have direct effect on the outcome.

In patients with uncontrolled T2DM, CVD contributes to nearly 52% of all-cause mortality. In general, T2DM is linked with a twofold increase in all-cause mortality and a threefold increase in cardiovascular mortality. Diabetes mellitus was alleged to be equivalent to coronary artery disease (Rajbhandari et al. 2021). Type 2 diabetes mellitus is also associated with heart failure, retinopathy, peripheral artery disease, stroke, neuropathy and nephropathy (Cade 2008).

### Limitations of the study

Multiple limitations emerged during the current study. Firstly, the small sample size could inhibit the generalisation of the findings in relation to the correlation between PWV, Aix and the study cytokines. Secondly, disease severity and the possible lack of compliance to treatment by study patients might have impacted the study outcome.

## Conclusion

For the first time, the effects of metformin alone and metformin plus glimepiride on arterial elasticity and pro-inflammatory markers in T2DM black South African participants have been investigated. Pulse wave velocity and Aix were directly correlated to pro-inflammatory cytokines (IL-1β, IL-6) and inversely correlated to anti-inflammatory cytokines (IL-1ra); however, metformin alone or in combination with glimepiride did not reduce pro-inflammatory cytokine levels and did not improve PWV and Aix, and therefore did not improve arterial elasticity over a period of 8 months of treatment. Due to the small sample size and the lack of adequate glucose level control in the study participants, a definite conclusion regarding the effects of metformin alone or in combination with glimepiride on arterial elasticity and pro-inflammatory cytokines in T2DM black South Africans cannot be made. Further investigations should be done on a larger sample size, with diabetes-controlled participants for a longer treatment period.

## References

[CIT0001] Alfadul, H., Sabico, S. & Al-Daghri, N.M., 2022, ‘The role of interleukin-1β in type 2 diabetes mellitus: A systematic review and meta-analysis’, *Front Endocrinol (Lausanne)* 13, 901616. 10.3389/fendo.2022.901616 35966098 PMC9363617

[CIT0002] Araki, T., Emoto, M., Teramura, M., Yokoyama, H., Mori, K., Hatsuda, S. et al., 2006, ‘Effect of adiponectin on carotid arterial stiffness in type 2 diabetic patients treated with pioglitazone and metformin’, *Metabolism* 55(8), 996–1001. 10.1016/j.metabol.2006.03.008 16839832

[CIT0003] Cade, W.T., 2008, ‘Diabetes-related microvascular and macrovascular diseases in the physical therapy setting’, *Physical Therapy* 88(11), 1322–1335. 10.2522/ptj.20080008 18801863 PMC2579903

[CIT0004] Chang, S., Kim, J., Sohn, T., Son, H. & Lee, J., 2018, ‘Effects of glucose control on arterial stiffness in patients with type 2 diabetes mellitus and hypertension: An observational study’, *Journal of International Medical Research* 46(1), 284–292. 10.1177/0300060517722697 28835148 PMC6011304

[CIT0005] Cruz, N.G., Sousa, L.P., Sousa, M.O., Pietrani, N.T., Fernandes, A.P. & Gomes, K.B., 2013, ‘The linkage between inflammation and Type 2 diabetes mellitus’, *Diabetes Research and Clinical Practice* 99(2), 85–92. 10.1016/j.diabres.2012.09.003 23245808

[CIT0006] Daryabor, G., Atashzar, M.R., Kabelitz, D., Meri, S. & Kalantar, K., 2020, ‘The effects of type 2 diabetes mellitus on organ metabolism and the immune system’, *Frontiers in Immunology* 11, 1582. 10.3389/fimmu.2020.01582 32793223 PMC7387426

[CIT0007] Dietrich, T., Schaefer-Graf, U., Fleck, E. & Graf, K., 2010, ‘Aortic stiffness, impaired fasting glucose, and aging’, *Hypertension* 55(1), 18–20. 10.1161/HYPERTENSIONAHA.109.135897 19933926

[CIT0008] Driessen, J.H.M., De Vries, F., Van Onzenoort, H.A.W., Schram, M.T., Van der Kallen, C., Reesink, K.D. et al., 2019, ‘Metformin use in type 2 diabetic patients is not associated with lower arterial stiffness the Maastricht Study’, *Journal of Hypertension* 37(2), 365–371. 10.1097/HJH.0000000000001892 30640873

[CIT0009] Ellulu, M.S. & Samouda, H., 2022, ‘Clinical and biological risk factors associated with inflammation in patients with type 2 diabetes mellitus’, *BMC Endocrine Disorders* 22, 16. 10.1186/s12902-021-00925-0 34991564 PMC8740444

[CIT0010] Franklin, S., 2008, ‘Beyond blood pressure: Arterial stiffness as a new biomarker of cardiovascular disease’, *Journal of the American Society of Hypertension* 2(3), 140–151. 10.1016/j.jash.2007.09.002 20409896

[CIT0011] Furmanova, O., Muz, N., Popova, V., Sayenko, Y., Orlenko, V., Ivas’kiva, R. et al., 2019, ‘Proinflammatory cytokines as biomarkers of effective metformin therapy in patients with type 2 diabetes’, *Diabetologia* 62(suppl 1), S25–S25.

[CIT0012] Hyun, B., Shin, S., Lee, A., Lee, S., Song, Y., Ha, N.J. et al., 2013, ‘Metformin down-regulates TNF-α secretion via suppression of scavenger receptors in macrophages’, *Immune Network* 13(4), 123–132. 10.4110/in.2013.13.4.123 24009539 PMC3759709

[CIT0013] Isoda, K., Young, J.L., Zirlik, A., MacFarlane, L.A., Tsuboi, N., Gerdes, N. et al., 2006, ‘Metformin inhibits proinflammatory responses and nuclear factor-kappaB in human vascular wall cells’, *Arteriosclerosis, Thrombosis, and Vascular Biology* 26(3), 611–617. 10.1161/01.ATV.0000201938.78044.75 16385087

[CIT0014] Janner, J.H., Godtfredsen, N.S., Ladelund, S., Vestbo, J. & Prescott, E., 2012, ‘The association between aortic augmentation index and cardiovascular risk factors in a large unselected population’, *Journal of Human Hypertension* 26(8), 476–484. 10.1038/jhh.2011.59 21654851

[CIT0015] Juan, L., Minglian, H. & Xingping, S., 2014, ‘The association of oxidative stress and pro-inflammatory cytokines in diabetic patients with hyperglycemic crisis’, *Journal of Diabetes and its Complications* 28(5), 662–666. 10.1016/j.jdiacomp.2014.06.008 25044235

[CIT0016] Kang, W., Wang, T., Hu, Z., Liu, F., Sun, Y. & Ge, S., 2017, ‘Metformin inhibits porphyromonas gingivalis lipopolysaccharide-influenced inflammatory response in human gingival fibroblasts via regulating activating transcription factor-3 expression’, *Journal of Periodontology* 88(10), e169–e178. 10.1902/jop.2017.170168 28548885

[CIT0017] Karbalaee-Hasani, A., Khadive, T., Eskandari, M., Shahidi, S., Mosavi, M., Nejadebrahimi, Z. et al., 2021, ‘Effect of metformin on circulating levels of inflammatory markers in patients with type 2 Diabetes: A systematic review and meta-analysis of randomized controlled trials’, *Annals of Pharmacotherapy* 55(9), 1096–1109. 10.1177/1060028020985303 33412927

[CIT0018] Lee, H.-Y. & Oh, B.-H., 2010, ‘Aging and arterial stiffness’, *Circulation Journal* 74(11), 2257–2262. 10.1253/circj.CJ-10-0910 20962429

[CIT0019] Lunder, M., Janić, M. & Šabovič, M., 2021, ‘Treating arterial ageing in patients with diabetes: From mechanisms to effective drugs’, *International Journal of Molecular Sciences* 22(6), 2796. 10.3390/ijms22062796 33801956 PMC8001638

[CIT0020] Matsumoto, K., Yasunori, S., Yasuyo, A., Tan, T., Yukitaka, Y. et al., 2004, ‘Metformin attenuates progression of carotid arterial wall thickness in patients with type 2 diabetes’, *Diabetes Research and Clinical Practice* 64(3), 225–228. 10.1016/j.diabres.2003.11.007 15126012

[CIT0021] Misra, D.P., Das, S. & Sahu, P.K., 2012, ‘Prevalence of inflammatory markers (high-sensitivity C-reactive protein, nuclear factor-κB, and adiponectin) in Indian patients with type 2 diabetes mellitus with and without macrovascular complications’, *Metabolic Syndrome and Related Disorders* 10(3), 209–213. 10.1089/met.2011.0044 22316266

[CIT0022] Oh, S., 2018, ‘Yuong. Arterial stiffness and hypertension’, *Clinical Hypertension* 24(17), 1. 10.1186/s40885-018-0102-8 30519485 PMC6271566

[CIT0023] Osuch, E., Du Plooy, W.J., Du Plooy, S.H. & Böhmer, L., 2012, ‘Effects of perindopril on pulse-wave velocity and endothilin-1 in black hypertensive patients’, *Cardiovascular Journal of Africa* 23(7), 396–399. 10.5830/CVJA-2012-043 22914998 PMC3721803

[CIT0024] Pickup, J.C., 2004, ‘Inflammation and activated innate immunity in the pathogenesis of type 2 diabetes’, *Diabetes Care* 27(3), 813–823. 10.2337/diacare.27.3.813 14988310

[CIT0025] Rajbhandari, J., Fernandez, C.J., Agarwal, M., Yeap, B.X.Y. & Pappachan, J.M., 2021, ‘Diabetic heart disease: A clinical update’, *World Journal of Diabetes* 12(4), 383–406. 10.4239/wjd.v12.i4.383 33889286 PMC8040078

[CIT0026] Rasakanya, T.L. & Osuch, E., 2023, ‘Arterial stiffness assessment in obese black South African patients’, *Cardiovascular Journal of Africa* 13(34), 1–5. 10.5830/CVJA-2022-064 36809558

[CIT0027] Saisho, Y., 2015, ‘Metformin and inflammation: Its potential beyond glucoselowering effect’, *Endocrine, Metabolic & Immune Disorders Drug Targets* 15(3), 196–205. 10.2174/1871530315666150316124019 25772174

[CIT0028] Shargorodsky, M., Omelchenko, E., Matas, Z., Boaz, M. & Gavish, D., 2012, ‘Relation between augmentation index and adiponectin during one-year metformin treatment for nonalcoholic steatohepatosis: Effects beyond glucose lowering?’, *Cardiovascular Diabetology* 11, 61. 10.1186/1475-2840-11-61 22676459 PMC3471007

[CIT0029] Takemura, Y., Osuga, Y., Yoshino, O., Hasegawa, A., Hirata, T., Hirota, Y. et al., 2007, ‘Metformin suppresses interleukin (IL)-1beta-induced IL-8 production, aromatase activation, and proliferation of endometriotic stromal cells’, *Journal of Clinical Endocrinology and Metabolism* 92(8), 3213–3218. 10.1210/jc.2006-2486 17504902

[CIT0030] Van Bortel, L.M., Laurent, S., Boutouyrie, P., Chowienczyk, P., Cruickshank, J.K., De Backer, T. et al., 2012, ‘Expert consensus document on the measurement of aortic stiffness in daily practice using carotid-femoral pulse wave velocity’, *Journal of Hypertension* 30(3), 445–448. 10.1097/HJH.0b013e32834fa8b0 22278144

[CIT0031] Wilkinson, I.B., Prasad, K., Hall, I.R., Thomas, A., MacCallum, H., Webb, D.J. et al., 2002, ‘Increased central pulse pressure and augmentation index in subjects with hypercholesterolemia’, *Journal of the American College of Cardiology* 39(6), 1005–1011. 10.1016/S0735-1097(02)01723-0 11897443

[CIT0032] Zak, K.P., Furmanov, O.V., Popov, V.V. & Sayenko, Ya.A., 2020, ‘The content of pro-inflammatory cytokines IL -1β, IL -6, IL -17A and TNF α in the blood of patients with type 2 diabetes after therapy with metformin’, *Ukrainian Biochemical Journal* 92(6), 105–112. 10.15407/ubj92.06.105

[CIT0033] Zheng, Z., Chen, H., Li, J., Li, T., Zheng, B, Zheng, Y. et al., 2012, ‘Sirtuin 1-mediated cellular metabolic memory of high glucose via the LKB1/AMPK/ROS pathway and therapeutic effects of metformin’, *Diabetes* 61(1), 217–228. 10.2337/db11-0416 22124463 PMC3237662

